# X-RAY AND MOLECULAR IMAGING DURING PREGNANCY AND BREASTFEEDING—WHEN SHOULD WE BE WORRIED?

**DOI:** 10.1093/rpd/ncab041

**Published:** 2021-04-14

**Authors:** Sören Mattsson, Sigrid Leide-Svegborn, Martin Andersson

**Affiliations:** Medical Radiation Physics Malmö, Department of Translational Medicine, Lund University, Malmö, SE-205 02 Malmö, Sweden; Medical Radiation Physics Malmö, Department of Translational Medicine, Lund University, Malmö, SE-205 02 Malmö, Sweden; Radiation Physics, Department of Hematology, Oncology and Radiation Physics, Skåne University Hospital, Malmö, SE-205 02 Malmö, Sweden; Medical Radiation Physics Malmö, Department of Translational Medicine, Lund University, Malmö, SE-205 02 Malmö, Sweden; Department of Radiation Physics, Institute of Clinical Sciences, Sahlgrenska Cancer Center, Sahlgrenska Academy, University of Gothenburg, Gothenburg, SE-413 45 Gothenburg, Sweden

## Abstract

Some of the ethically most sensitive issues in radiation protection arise at imaging of pregnant—and potentially pregnant—patients and of newborn.

This article reviews the current literature and recommendations on imaging during pregnancy and breastfeeding. Risks related to alternative non-ionizing radiation methods are also considered.

With few exceptions, exposure of the fetus through radiography, computed tomography (CT) and nuclear medicine imaging can be limited to safe levels, although studies such as abdominal-pelvic CT cannot avoid significant exposure to fetuses. Eight to 10 weeks post-conception, the fetus has a thyroid which starts to concentrate iodide having crossed the placenta barrier resulting in unacceptably high doses to the fetal thyroid after administration of ^131^I- and even ^123^I-iodide and other radiopharmaceuticals with a high content of free radioiodine.

Many radiopharmaceuticals are excreted through breast milk. Breastfeeding interruption recommendations should be followed to keep the effective dose to the infant below 1 mSv.

## INTRODUCTION

Some of the most ethically sensitive issues in radiation protection arise at radiological imaging of pregnant—and potentially pregnant—patients and of newborns. Therefore, it is important to periodically update available information about fetal doses and doses to e.g. breastfed infants and the associated risks.

The use of radiography, computed tomography (CT) and nuclear medicine as well as ultrasound (US) and magnetic resonance imaging (MRI) is today so integrated in healthcare that women with recognized or unknown pregnancies are likely to be examined by any of these methods. Personnel in healthcare frequently face the dilemma of exposing pregnant or possibly pregnant patients to radiation from diagnostic examinations^(1)^. Irradiation of the fetus occurs more commonly than suspected^(2)^, and one should be aware of the implicated risks. The number of investigations in pregnancy using ionising radiation has increased, more for CT than for the other methods. Lack of knowledge about the health consequences may lead to unjustified anxiety among patients, healthcare professionals and the general public and sometimes result in unnecessary avoidance of essential diagnostic investigations and delayed diagnosis. This applies to both X-rays and radiopharmaceuticals (as well as to contrast agents for X-rays and MRI). The most correctly performed diagnostic procedures are medically appropriate and the risk of radiation to the embryo or fetus is minimal. There are however exceptions—occasions when the fetal exposure is inappropriate and the embryo or fetus may be at increased risk. Still, higher doses such as those from therapeutic procedures can result in very significant birth defects.

The aim of this paper is to review available literature and recommendations on radiation protection at diagnostic imaging during pregnancy and breastfeeding.

## IDENTIFICATION OF PREGNANCY AND BREASTFEEDING

To be able to take pregnancy in women of reproductive age (14–55 years) into consideration, it is necessary to identify who is pregnant. Identification of pregnant women can be done through questions in the invitation to the examination, by information posters in the waiting room and repeated questions shortly before the investigation whether the woman is pregnant or could be pregnant. In this context, it is important to consider cultural and generational differences in attitudes to discussions about pregnancy. If necessary, a pregnancy test should be done.

Current recommendations do not take into account the ‘safe’ period during the menstrual cycle. The former concept of the ‘10-day rule’ (which means that whenever possible, one should confine the radiological examination of the lower abdomen and pelvis to the 10-day interval following the onset of menstruation) has been considered obsolete for several years. It is the responsibility of the healthcare system to investigate whether a woman in reproductive age may be pregnant and a responsibility of the patient to inform if she may be pregnant. If this is the case, the investigation may be postponed until after birth or the investigation changed to another method, which is not based on ionizing radiation. Diagnostic imaging should only be performed during pregnancy with an understanding of the risks and benefits of the mother and fetus, the comparative advantages of different modalities and the unique anatomical and physiological issues associated with pregnancy^(3–5)^. The woman has the right to know the risk of the examination or treatment for both her own sake and the fetus’ sake. Regarding the identification of breastfeeding women, similar routines in the form of repeated questions and information posters should be used to identify who is breastfeeding before a nuclear medicine investigation. Also in this case, the examination may be postponed until after breastfeeding has been terminated intentionally and planned, or the investigation can be changed to a method other than a nuclear medicine one.

**Figure 1:**
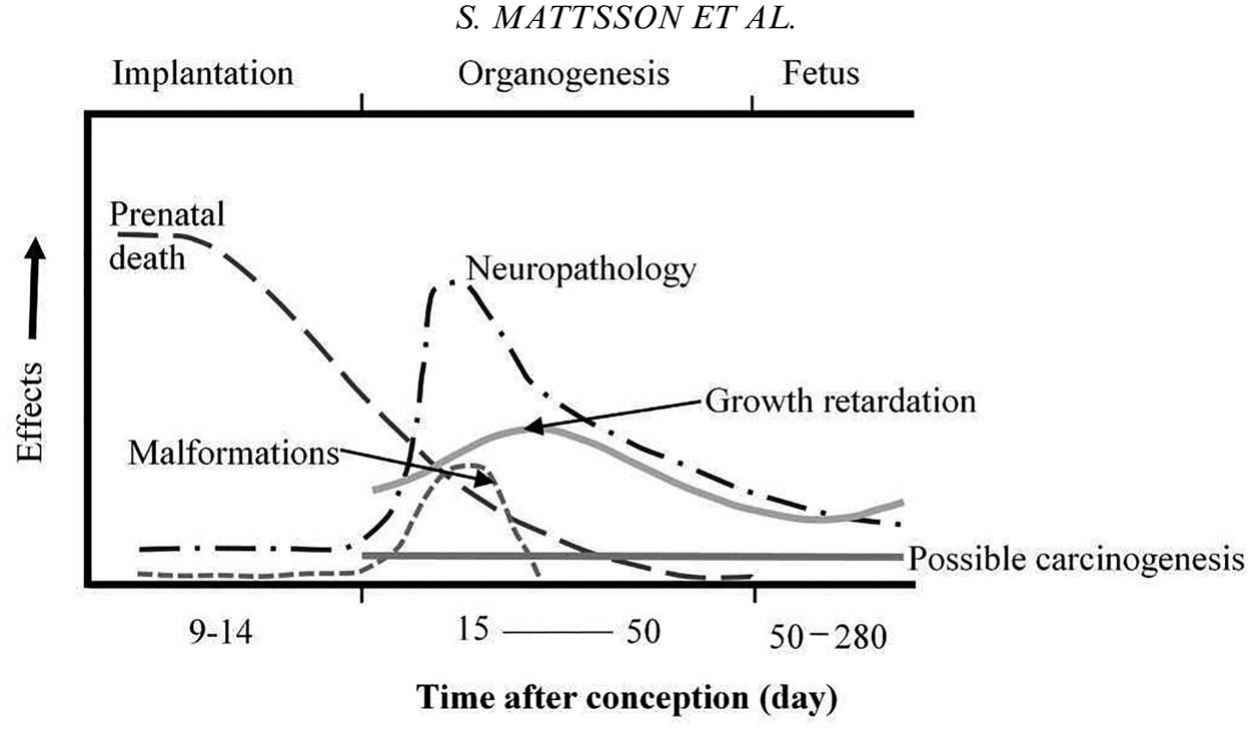
The figure shows the deterministic effects in the form of prenatal death during the implantation period and cell death during the organogenesis—resulting in growth retardation, malformations and neurological effects (microcephaly, intellectual or developmental disability). The risk of each effect depends on the gestational age at the time of exposure and the absorbed dose, free after Vipul and Sen^(45)^. The increased risk of cancer (as a child or as an adult) after exposure at various times after exposure is also indicated.

## RADIATION RISKS DURING THE FETAL PERIOD AND DURING THE FIRST YEAR OF LIFE

There are two types of effects and risks resulting from ionizing radiation: (1) short-term deterministic risks and (2) long-term stochastic risks. Deterministic short-term effects in the form of prenatal death during the implantation period and cell death during the organogenesis—resulting in growth retardation, malformations and neurological effects (microcephaly, intellectual or developmental disability). The risk of each effect depends on the gestational age at the time of exposure (Figure 1), and the absorbed dose to the fetus. All observations of significant IQ reduction and severe mental retardation relate to fetal doses of 500 mGy or higher and at high dose rates. These effects are not expected to occur at an absorbed dose under 100 mGy^(6,7)^, which is an important observation for X-ray and nuclear medicine imaging.

On the other hand, we must consider the increased risk of cancer (as a child or as an adult)^(8)^. This together with hereditary effects, represents the long-term stochastic risks of ionizing radiation. The increased risk of malignancy in children, including leukemia and lymphoma, is estimated to 10^−4^/mGy after exposure *in utero*^(9)^. The risk to the embryo/fetus depends on: the exposed part of the mother’s body, the stage of pregnancy and the absorbed dose. About 1 in 500 children develops cancer before the age of 14 years (in the absence of extra radiation as a fetus)^(10)^. For a fetus exposed to one of the highest diagnostic doses after the first 4 weeks post-conception, the risk of childhood cancer might increase to 3 out of 500. The risk for hereditary effects is small in comparison to the cancer risk for the exposed individual. The risk for the newborn is of the same order of magnitude as for the fetus during the third trimester. A 10–20 mGy fetal exposure may increase the risk of leukemia by a factor of 1.5–2.0 over a background of ~1 in 3000. There are a number of publications with more details on risks after irradiation *in utero*^(11,12)^ and as a young child^(13)^. It should be added that there is a continuously ongoing debate about the accuracy and uncertainty of today’s risk estimates^(14,15)^.

## FETAL DOSES IN RADIOGRAPHY, FLUOROSCOPY, CT AND INTERVENTIONAL RADIOLOGY

**Figure 2:**
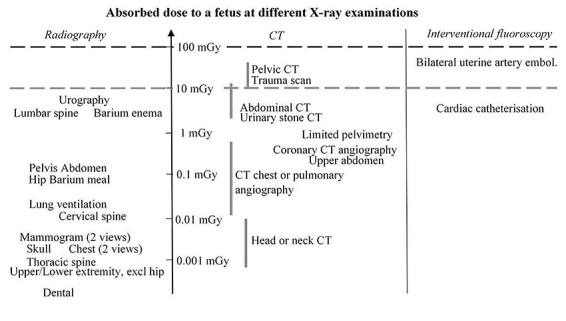
Mean absorbed dose to a fetus for planar X-ray imaging, for CT investigations and for some X-ray guided interventional procedures^(^6^^,^46–52)^. The absorbed dose varies with gestational age, maternal physical constitution and image acquisition parameters (as indicated for some of the CT-investigations). The horizontal lines mark the 100-mGy level, which is considered as a threshold for short-term deterministic effects and the 10-mGy level, which is the dose level where we have to start to consider the increased risk of cancer.

The absorbed dose to a fetus varies significantly for the different X-ray examinations (Figure 2). The figure shows the mean absorbed dose to a fetus in X-ray imaging; for planar imaging, for CT investigations and for some interventional procedures. The dose varies with gestational age, the mother’s physical constitution and parameters for image acquisition. The horizontal lines mark the 100-mGy level, which is considered a threshold for short-term deterministic effects during organogenesis and the 10-mGy level, which is the dose level at which we must begin to consider the increased risk of cancer. The highest doses come from CT investigations in the pelvic and abdominal regions and from some specific interventional procedures. Conventional X-ray imaging gives low to very low fetal doses.

### Action plans for radiological examinations when pregnancy is confirmed or probable

If pregnancy is established or probable and the fetus will be in the field of view, the justification of the investigation should be reviewed. If still justified, particularly careful optimization of the imaging conditions should be done.

In some studies, it has been shown that fetal doses from scattered radiation can be reduced with the help of shielding; for example, lead apron wrapped around the mother’s abdomen during mammography reduces the already very low doses to the uterus with ~50%^(16,17)^. At other investigations, like pulmonary angiography, shielding only modestly reduces fetal radiation dose, but may compromise the automatic exposure control, possibly increasing the maternal and fetal radiation dose. Shortening the scan is beneficial, assuming anatomical coverage is secured^(18)^.

### Interventional procedures

When an interventional procedure is required, US should be used for guidance whenever possible. If the use of X-rays is unavoidable, exposure should be reduced by minimizing fluoroscopy time, reducing the number of images, using the lowest possible frame rate, optimizing collimation, using image hold, etc. In a valuable guideline intended to assist interventionists and their staff in managing and counseling pregnant patients who need fluoroscopically or CT-guided interventional procedures, Dauer *et al.*^(19)^ discuss radiation management for interventions using fluoroscopic or CT guidance during pregnancy.

### CT

Estimated exposure to fetal radiation can be limited to safe levels for most diagnostic procedures, although studies such as abdominal-pelvic CT cannot avoid significant exposure to fetuses and should therefore, if possible, be avoided.

If the exposure is medically unavoidable, dose reduction strategies should be used: lowering of tube potential based on the patient’s weight, decreasing the tube current-time product, limiting the scan length, increasing pitch, limiting the number of scans, using automated exposure control, tube potential selection and iterative image reconstruction. The most common indications for acute CT during pregnancy are^(20)^: (1) appendicitis—for first and second trimester pregnancies US and/or MR should be performed before obtaining a CT; (2) pulmonary embolism; (3) renal colic—US is the first study of choice; (4) trauma—US may be sufficient for the initial imaging evaluation of a pregnant patient who has sustained trauma, but CT should be performed if serious injury is suspected; (5) CT of other parts of the body than abdomen and pelvis, constitute negligible risks: CT is even the preferred method for imaging of suspected pulmonary embolism in pregnancy in many countries. In our opinion, an optimized lung perfusion scintigraphy should be the first choice to further reduce the absorbed dose to the fetus as well as to the mother (especially to the breast tissue)^(21)^. The fetal dose varies between the two modalities depending on the age of the fetus and the imaging protocols used. The absorbed dose to a fetus in the first trimester is significantly lower for CT than for lung scintigraphy, in the second trimester the doses are comparable and in the third trimester the dose to the fetus is somewhat higher for a CT-procedure. The dose to the breasts of the mother is however considerably higher for the CT-procedure than for the scintigraphy resulting in a higher effective dose. Pelvimetry can be performed by low dose CT (or by MRI).

### Risks from X-ray contrast agents

Non-ionic iodinated agents are (like gadolinium-based agents for MRI) water soluble and can cross the placental barrier. In the fetal circulation, molecules of iodinated contrast material can pass through the kidneys and make their way into the amniotic fluid via the urine. When the fetus swallows amniotic fluid, a small amount of contrast material can enter the fetal gastrointestinal tract. An additional small amount of contrast medium may pass directly from the mother’s blood into the amniotic fluid, be swallowed by the fetus and reach the fetal gastrointestinal tract.

For iodinated X-ray contrast material in pregnancy, no mutagenic or teratogenic effects have been described. The main detrimental effect of iodine-based compounds is their potential impact on the newborn thyroid gland^(22)^. Therefore, after administration of iodinated agents to the mother during pregnancy, thyroid function should be monitored in neonates during the first week^(23)^. The use of iodinated contrast agents is generally safe during pregnancy. Nevertheless, these should be used with caution and should only be administered when the clinical situation clearly requires it.

## FETAL DOSES FROM DIAGNOSTIC EXAMINATIONS IN NUCLEAR MEDICINE

### Radiopharmaceuticals other than those labelled with radioactive iodine

**Figure 3:**
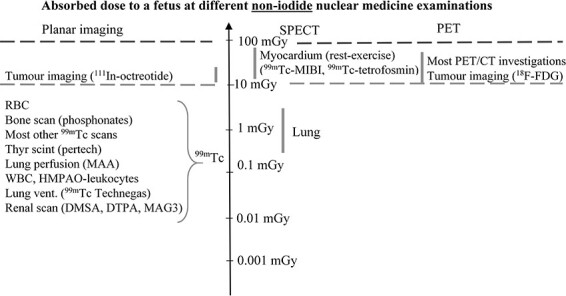
Mean absorbed dose to a fetus for a number of nuclear medicine investigations using radiopharmaceuticals other than those labelled with radioactive iodine^(^^6^^,^^39^^,^^48^^,^^50^^)^. The estimated absorbed dose for a specific radiopharmaceutical varies according to administered activity, method of dose estimation, and patient-dependent factors (e.g. stage of pregnancy, weight and body habitus). MAA - macro-aggregated albumin, HMPAO - hexamethylpropyleneamineoxine, DMSA - dimercaptosuccinic acid, DTPA - diethylenetriaminepentaacetic acid, MAG3 - mercaptoacetyl triglycine, MIBI - 2-methoxy-isobutyl-isonitrile.

The absorbed dose to a fetus from a number of nuclear medicine diagnostic procedures with radiopharmaceuticals other than those labelled with radioactive iodine is shown in Figure 3. For planar imaging using ^99m^Tc-labelled substances, doses are low. The highest fetal doses come from SPECT and PET investigations. Today a low dose CT is often performed with PET and increasingly with SPECT. In this way the CT dose component can be reduced with a factor of 2–4^(24,25)^ in comparison to a standard diagnostic CT investigation.

High doses are also received from some specific planar tumor imaging. This can partly be explained by the fact that for these investigations higher activity (MBq) is needed in order to get an acceptable image quality. Dose calculations for children are still generally based on biokinetic data for adults. Very few biokinetic data have been collected for children of various ages. The biological distribution, uptake and retention of radiopharmaceuticals may vary considerably throughout childhood and need to be taken into account.

### Radiopharmaceuticals labelled with radioactive iodine

**Figure 4:**
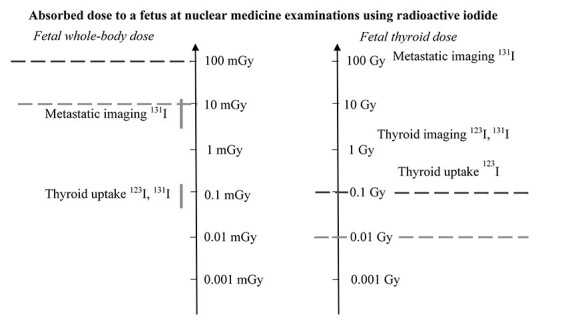
Mean absorbed dose to a fetus for a number of nuclear medicine investigations using radioactive iodide. The fetal thyroid doses are much higher than fetal whole-body dose, e.g. 5–15 mGy/MBq for ^123^I and 500–1100 mGy/MBq for ^131^I if the thyroid is present and accumulating iodide^(^^6^^,^^39^^,^^48^^,^^50^^)^.

For investigations using radioactive iodide, the situation is more problematic as can be seen in Figure 4. Eight to 10 weeks after conception (10–12 weeks’ gestational age), the fetal thyroid starts to accumulate iodide that has crossed the placenta barrier^(6,26)^. The fetal thyroid dose will be much higher than the fetal whole-body dose, e.g. 2.7–6.4 mGy/MBq for ^123^I and 230–580 mGy/MBq (given to the mother) for ^131^I if the thyroid is present and accumulating iodide^(27)^. The consequences are that in spite of a low whole body fetal dose, the dose to the fetal thyroid is so high that the function of the thyroid gland may be affected despite a low fetal dose throughout the body.

High fetal thyroid doses can result in hypothyroidism with consequences for the thyroid hormone production and for the development of the fetus, dwarfism and also an increased risk for thyroid cancer. If pregnancy is discovered within 12 h of radioiodide administration, rapid and repeated oral administration of stable potassium iodide (60–130 mg) to the mother may reduce the fetal thyroid dose.

For therapy with ^131^I, pregnancy is an absolute contraindication. Fetal thyroid doses of several 100 Gy can be reached^(28,29)^, resulting in total elimination of the thyroid function and thus risk for very serious effects on the fetus.

Radiopharmaceuticals labelled with ^123^I are frequently used in diagnostic nuclear medicine, e.g. ^123^I-mIBG and ^123^I-ioflupane. The ^123^I used is nowadays, typically produced through irradiation of highly enriched xenon-124 gas which makes the ^123^I end product ultra-pure^(30)^. Nevertheless, there is a slight probability of impurities, such as ^124^I and ^125^I, ~0.3% of the total activity^(31)^.

The radiochemical purity of ^123^I-mIBG and ^123^I-ioflupane preparations is high, although a fraction of free ^123^I of ~2–3 and 6% for ^123^I-mIBG and ^123^I-ioflupane, respectively, can be expected according to the summary of product characteristics for each product. Routinely, thyroid blocking treatment is therefore carried out when performing these procedures, in order to minimize the uptake of free iodide in the thyroid. This will prevent the uptake of free iodide in the thyroid of a fetus/embryo and breastfed infant as well.

Assuming no placental cross-over of ^123^I-mIBG and ^123^I-ioflupane or of free ^123^I, the absorbed dose to the fetus from these radiopharmaceuticals is ~4 mGy per investigation with 200 MBq given to the mother.

### Action plans for diagnostic nuclear medicine when pregnancy is confirmed or probable

The problems are mainly due to radioactive iodide and to radiopharmaceuticals containing radioiodine. Most other diagnostic nuclear medicine procedures, except some SPECT and PET investigations, do not cause large fetal doses. For those with higher doses, fetal doses can be reduced using less activity and longer measurement time. For radiopharmaceuticals that are excreted through the kidneys, which applies to the majority of radiopharmaceuticals, the fetal dose can be reduced by encouraging the mother to drink more than usual (1–2 L of extra fluid) so that she can empty the bladder frequently for 1–2 days after the administration of the radiopharmaceutical.

## RISKS FROM ALTERNATIVE NON-IONISING RADIATION METHODS

### Ultrasound

Diagnostic US has been used during pregnancy for many years and there are no documented fetal effects of diagnostic US. It is generally considered safe when used appropriately. The lowest amount of US energy that provides an accurate assessment should be used. Therefore, US equipment constructed for use in obstetrics does not produce the rise in temperature delivered by equipment for non-obstetric use. Like other imaging modalities, US should be used prudently and only when its use is expected to answer a relevant clinical question or otherwise provide medical benefit to the patient^(32)^.

### MRI

With the increased awareness of the negative effects of ionizing radiation to infants and children, there is an increasing interest of using MRI instead of over CT in pregnancy and for children. The previous recommendation was to avoid MRI during the first trimester as there were no prior controlled studies on its safety in the first trimester, when the fetus forms its major organs and body structures. Today MRI is considered to pose no known risk to the fetus^(33,34)^. However, caution is advised, and risks and benefits must always be considered before evaluating a pregnant patient with MR imaging.

Gadolinium MRI at any time during pregnancy was reported to be associated with an increased risk of a broad set of rheumatologically, inflammatory, or infiltrative skin conditions and for stillbirth or neonatal death^(35)^. Gadolinium contrast should therefore be avoided when examining a pregnant patient and only be used if their use is considered critical and the potential benefits justify the potential unknown risk to the fetus^(33,36)^.

## BREASTFEEDING AFTER ADMINISTRATION OF DIAGNOSTIC RADIOPHARMACEUTICALS, X-RAY AND MR-CONTRAST AGENTS

### Radiopharmaceuticals

Administration of radiopharmaceuticals to breastfeeding mothers is generally avoided because the activity is secreted into the breast milk, resulting in unnecessary radiation exposure of the infant who ingests the milk. In those circumstances when a nuclear medicine procedure of a nursing mother is vital for the mother, it may be necessary to interrupt breastfeeding, either temporarily or permanently. Breastfeeding is very important for the infant as well as for the mother^(37,38)^ and should not be interrupted if not absolutely necessary.

To keep the effective dose to the child from ingestion of the breastmilk below 1 mSv, breastfeeding should be suspended as follows^(39,40)^:

- Completely after ^131^I therapy and all other therapies with radiopharmaceuticals.
- 3 weeks after ^131,125,123^I-, ^75^Se-, ^67^Ga- and ^22^Na-compounds except as indicated below.
- 48 h after ^201^Tl-chloride.
- 12 h after ^131,125,123^I-hippurate and ^99m^Tc-pertechnetate, -macro-aggregated albumin (MAA), -microspheres, -red blood cells (RBC) *(in vivo*) and -leukocytes.
- Not necessary after other ^99m^Tc-compounds (-diisopropyl iminodiacetic acid (DISDA), -diethylenetriaminepentaacetic acid (DTPA), -ethylenedicysteine diester (ECD), phosphonates, RBC *(in vitro*), DTPA), ^11^C-, ^13^N-, ^15^O-labelled substances, ^18^F-FDG, ^51^Cr-ethyl-enediaminetetraacetic acid (EDTA), ^81m^Kr-gas, ^111^In-octreotide, ^111^In-leukocytes and ^133^Xe-gas.

The International Atomic Energy Agency (IAEA) has also published recommendations on breastfeeding interruption after a nuclear medicine procedure^(41)^. They do not differ significantly from those of the International Commission on Radiological Protection (ICRP) apart from some specific radiopharmaceuticals. The IAEA recommendations include also external irradiation of the infant due to close contact with the mother. This is significant in the case of ^18^F-FDG, where interruption in breastfeeding is not necessary according to the list above but where IAEA recommends an interruption period of 4 h in order to certify that the mother and infant is not close. For most ^99m^Tc-labelled substances it is not necessary to interrupt breastfeeding, based upon the total fraction of the activity administered to the mother that is excreted into the breast milk and ingested by the infant. The risk of free ^99m^Tc-pertechnetate in the preparation justifies however a 4-h interruption during which one meal is discarded, to be on the safe side. For newer radiopharmaceuticals where no information on radionuclide excretion in breast milk have been published, several consecutive samples of breast milk have to be collected. The activity concentration in these samples has to be measured and the cumulated activity determined. Based upon this information and biokinetic data on the radiopharmaceutical within the body the absorbed dose to various organs and tissues and the effective dose can be calculated.

### No breastfeeding after administration of therapeutic radiopharmaceuticals

Breastfeeding has to be stopped permanently after all forms of therapy with radiopharmaceuticals. Women who have to undergo therapy with radioiodine should stop breastfeeding 2–3 weeks before receiving therapy. This is because breast tissue that is producing milk will receive an unnecessary high dose due to accumulation of activity in the breasts, higher than the breast tissue of a woman who is not breastfeeding.

### X-ray and MR contrast agents

Only a small percentage of iodinated contrast material or gallium-based contrast agents is excreted in breast milk and absorbed by the infant, and there have been no reported cases of direct toxicity, allergic sensitivity, or reaction to these agents. Breast feeding can therefore be continued without interruption after administration of iodinated contrast or gadolinium contrast to a nursing patient^(^^22^^,^^23^^,^^33^^,^^36^^,^^42^^)^.

## TERMINATION OF PREGNANCY

Termination of pregnancy is an individual decision by the mother affected by many factors. Fetal doses below 100 mGy should not be considered a reason for terminating a pregnancy based on radiation risk. At fetal doses in excess of 500 mGy, there can be significant fetal damage, the magnitude and type of which is a function of dose and stage of pregnancy. At fetal doses between 100 and 500 mGy, decisions should be based upon individual circumstances after information about (1) risk of serious harm to the fetus and (2) increased risk of cancer later in life^(6)^.

## STAFF

Radiological protection of fetuses and breastfed children of occupationally exposed women in X-ray and nuclear medicine continues to be a topical issue due to the continuous change and advances of X-ray and molecular imaging. The high proportion of female workers in healthcare; among technologists, radiographers, physicians, medical physicists and biomedical engineers, emphasizes the need for a robust strategy regarding protection of fetuses and breastfed children in the healthcare sector^(43,44)^. It is advised that each facility sets up local guidelines and defines work activities that can be done without restrictions and those tasks that are not suitable to perform when pregnant or breastfeeding. Information about the necessity of declaring pregnancy or breastfeeding is another fundamental issue. This information has to be accompanied with information about what happens if declaring pregnancy or breastfeeding. In order to properly protect a fetus and a breastfed child, it should be made clear by management that workers will not be punished and that the facility is prepared to handle this important issue professionally. Once a pregnancy is confirmed the dose to the fetus must not exceed 1 mSv for the rest of the pregnancy^(6)^.

## SUMMARY AND CONCLUSION

For most medically appropriate and correctly performed diagnostic tests, the risk of embryos and fetuses is minimal. However, due to the possibility of higher fetal exposure in CT or radionuclide imaging, identification of pregnant and potentially pregnant women should be performed before performing radiodiagnostic examinations. If necessary, pregnancy tests should be performed. If pregnancy is confirmed or probable, non-ionising radiation-emitting imaging, such as MRI and US, should be considered first, when clinically appropriate. However, some pregnant women will still be faced with the decision to undergo CT or nuclear medicine imaging because the test is clinically justified. In such cases, careful optimization of the investigations should be done in order to minimize the dose to the fetus or infant and still obtain acceptable image quality. There is also a need to reduce the anxiety that a mother experiences before and after birth. Many radiopharmaceuticals are excreted through breast milk. Breastfeeding interruption recommendations should be followed to keep the effective dose to the infant below 1 mSv. It is very important to permanently stop breastfeeding after therapy with all radiopharmaceuticals and also after diagnostic use of radioiodide and radioiodine with high content of free iodide.

## CONFLICT OF INTEREST STATEMENT

No conflict of interest was declared.
